# COVID-19 vaccines: their effectiveness against the severe acute respiratory syndrome coronavirus 2 (SARS-CoV-2) and its emerging variants

**DOI:** 10.1186/s42269-022-00787-z

**Published:** 2022-04-08

**Authors:** Rashed Noor, Saadia Shareen, Muntasir Billah

**Affiliations:** 1 Department of Life Sciences (DLS), School of Environment and Life Sciences (SELS), Independent University, Bangladesh (IUB), Plot 16, Block B, Aftabuddin Ahmed Road, Bashundhara, Dhaka, 1229 Bangladesh; 2 Department of Cardiology, Kolling Institute of Medical Research, Northern Sydney Local Health District, St Leonards, NSW 2065 Australia

**Keywords:** COVID-19, SARS-CoV-2, Vaccines, Variants, Mutations, Vaccine effectiveness, Neutralizing antibodies

## Abstract

**Background:**

The world has been suffering from the COVID-19 pandemic (officially declared by WHO in March 2020), caused by the severe acute respiratory β-coronavirus 2 (SARS-CoV-2) since the last week of December 2019. The disease was initially designated as a Public Health Emergency of International Concern on January 30, 2020. In order to protect the health of mass public, an array of research on drugs and vaccines against SARS-CoV-2 has been conducted globally. However, the emerging variants of SARS-CoV-2, i.e., Alpha (B.1.1.7), Beta (B.1.351), Gamma (P.1), and Delta (B.1.617.2) variants which evolved in late 2020 and the Omicron variant (B.1.1.529) which emerged in November 2021 along with its subvariant BA.2 which was first identified in India and South Africa in late December 2021, have raised the doubt about the efficiency of the currently used vaccines especially in terms of the consistent potential to produce neutralizing antibodies targeting the viral spike (S) protein.

**Main body of the abstract:**

The present review discussed the functional details of major vaccines regarding their efficiency against such variants during the pandemic. Overall, the mRNA vaccines have shown around 94% effectiveness; the adenovector vaccine showed approximately 70% efficacy, whereas Sputnik V vaccines showed around 92% effectiveness; the inactivated whole-virus vaccine CoronaVac/PiCoVacc and BBIBP-CorV showed a varying effectiveness of 65–86% according to the geographic locations; the subunit vaccine NVX-CoV2373 has shown 60–89% effectiveness along with the global regions against the wild-type SARS-CoV-2 strain. However, reduced effectiveness of these vaccines against the SARS-CoV-2 variants was noticed which is suggestive for the further administration of booster dose.

**Short conclusion:**

Maximum variants of SARS-CoV-2 emerged during the second wave of COVID-19; and extensive studies on the viral genomic sequences from all geographical locations around the world have been conducted by an array of groups to assess the possible occurrence of mutations(s) specially within the receptor binding domain of the viral spike (S) protein. Mutational similarities and the new or critical mutations within all variants have been clearly identified so far. The study of effectiveness of the currently used vaccines is also ongoing. The persistence of memory B cell action and the other immune components as well as the administration of booster dose is expected to mitigate the disease.

## Background

A cluster of patients with acute respiratory distress syndrome (ARDS) was reported in China on December 31, 2019, which was later confirmed as novel coronavirus on January 7, 2020 (World Health Organization 2020). The history of human coronavirus began in 1965 when Tyrrell and Bynoe found a virus named B814, obtained from respiratory tract of an adult with a common cold (Tyrrell and Bynoe 1966). While exploring the epidemiology of the human coronavirus, results from animal studies concluded that it caused disease in multiple animal species including rats, mice, chicken, turkeys, dogs, cats, rabbits and pigs (Tyrrell and Bynoe 1966). Given the enormous diversity of animal coronavirus, it was almost unsurprising with the emergence of new severe acute respiratory syndrome coronavirus (SARS-CoV) in 2002–2003 (Drosten et al. 2003; Ksiazek et al. 2003). Since 2005, four new coronaviruses have been discovered (Kahn and McIntosh 2005). The current COVID-19 pandemic marks almost the 7th incidence of coronavirus in the human history.

COVID-19 pandemic, caused by severe acute respiratory syndrome coronavirus 2 (SARS-CoV-2), has been the most devastating global public health crisis of this century, resulting in in 5,770,023 deaths out of 402,044,502 affected cases as of February 10, 2022 (WHO 2022). This is already well known that SARS-CoV-2, a member of the β-coronavirus genus, is closely related to the severe acute respiratory syndrome coronavirus-1 (SARS-CoV-1 which took place in 2002–2003) since they have around 79% genetic similarity between them and 50% genetic similarity with the Middle East respiratory coronavirus (MERS-CoV) which caused an endemic in 2012 (Noor 2021b).There are four genera of all coronaviruses, i.e., Alpha (α), Beta (β), Gamma (γ), and Delta (δ); the β-coronaviruses constitute the severe human coronaviruses SARS-CoV-2, SARS-CoV and MERS-CoV along with three other human coronaviruses which cause mild onset of the disease (Noor and Maniha 2020). Being the etiological agent of the present pandemic SARS-CoV-2 and the causative agent of the 2002–2003 epidemic, the SARS-CoV has been reported with 79% sequence homology between them, whereas SARS-CoV-2 and MERS-CoV have been noticed to possess only 50% homology, revealing SARS-CoV-2 as a significantly distant strain both from SARS-CoV-1 and from MERS-CoV (Noor and Maniha 2020). All these three coronaviruses exert the highly conserved genomic organization, and they employ the similar patterns of the life cycle steps within the host during their pathogenesis and expression of the corresponding virulence factors; however, the extent of infectivity has been markedly noticed to be fatal in case of SARS-CoV-2.

**Fig. 1:**
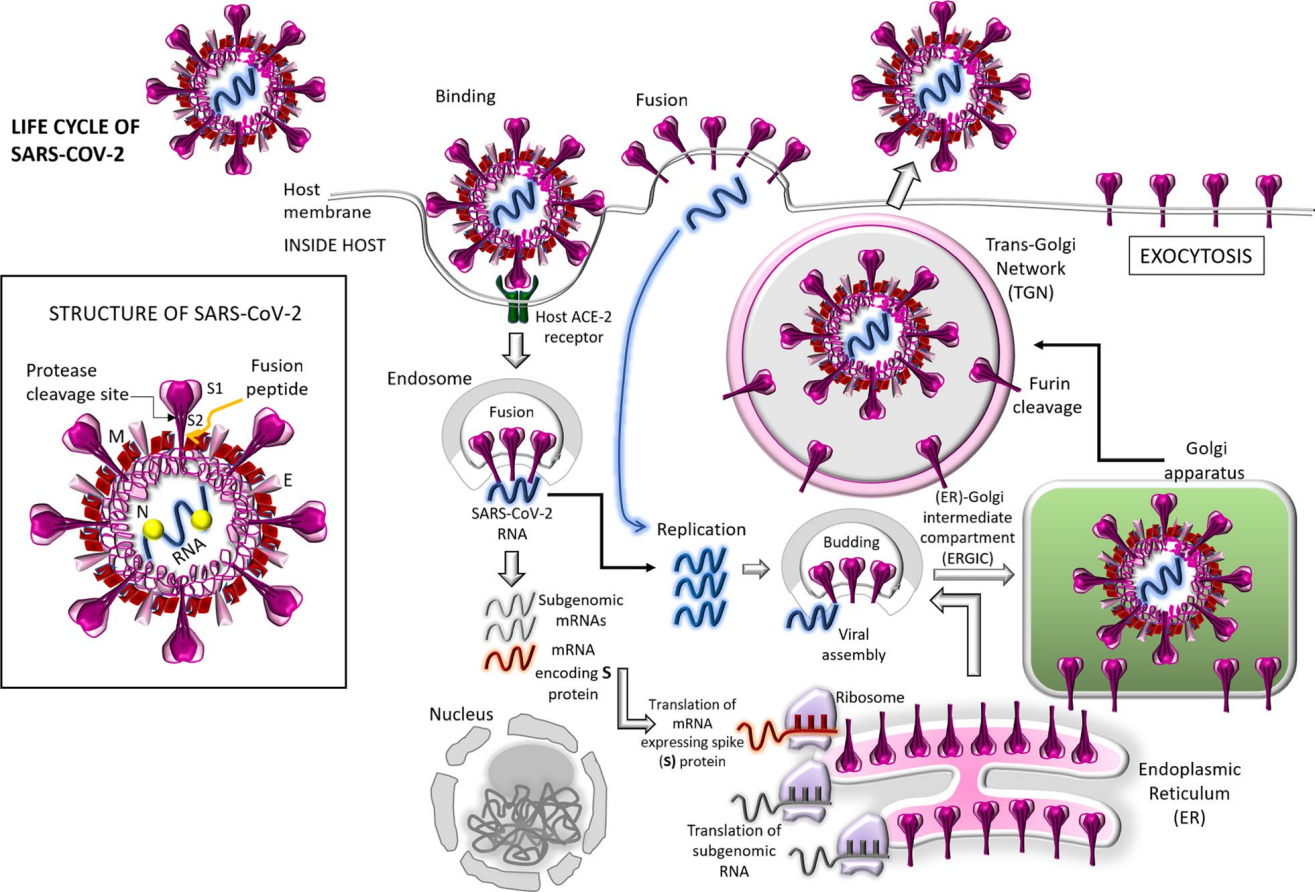
Life cycle of severe acute respiratory syndrome coronavirus-2 (SARS-CoV-2). In the inset, the structure of SARS-CoV-2 is shown. The release of viral RNA takes place upon the viral entry, followed by the replication of viral RNA leading to the formation of subgenomic RNAs of which one category may encode the viral spike (S) protein (Drosten et al. 2003; Ksiazek et al. 2003; Noor and Maniha 2020). After translation within the ribosomes, the newly synthesized S protein migrates to the lumen of endoplasmic reticulum (ER); and new virus particles generate through budding into the lumen of the ER-Golgi intermediate compartment (ERGIC) (Kahn and McIntosh 2005). With the action of exocytosis, the virions get released; and subsequently the S protein is matured into SI and S2 subunits in the trans-Golgi network (TGN) instigated by the cellular protease, furin (Kahn and McIntosh 2005)

**Fig. 2:**
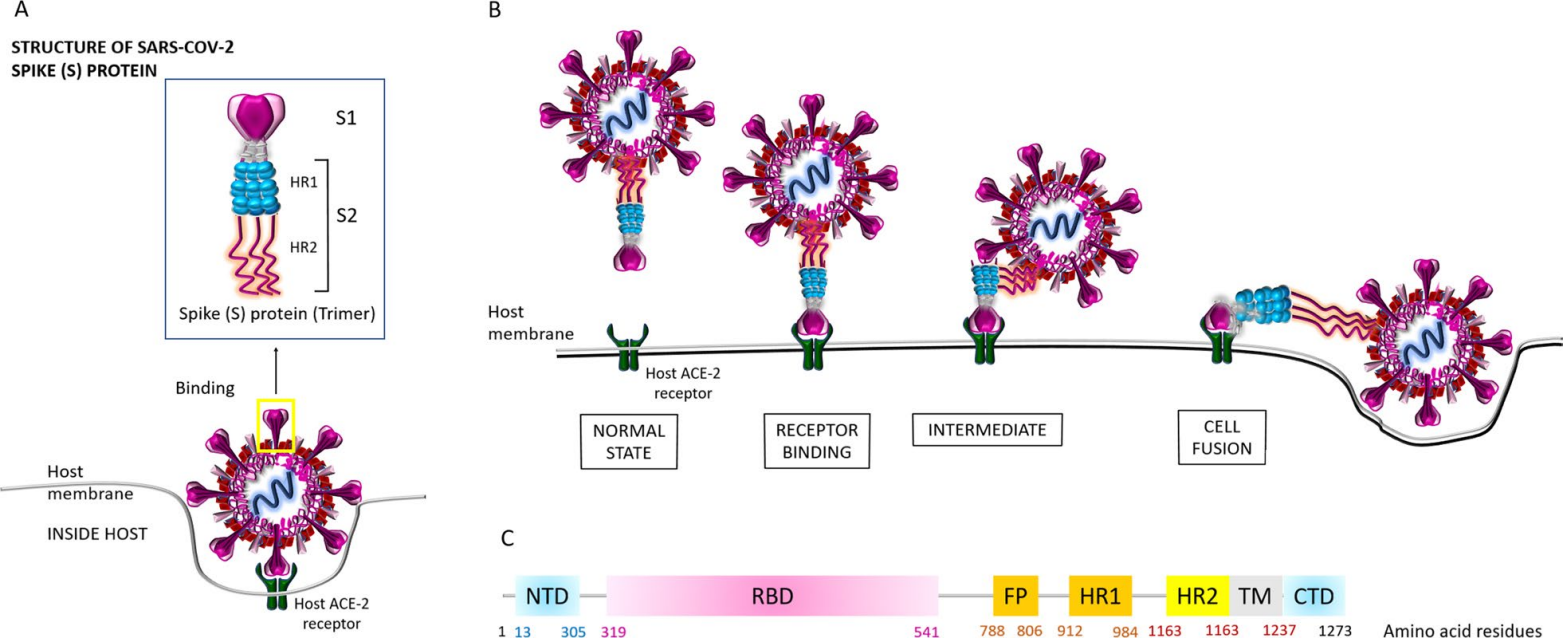
SARS-CoV-2 spike (S) protein structure. **A** The binding mechanism of the host ACE-2 receptor by the SARS-CoV-2 spike (S) protein which has 1273 amino acid residues (180–200 kDa) has been shown. S protein trimers are the crown-like halo structures surrounding the viral particle. The S1 and S2 subunits form the bulbous head and stalk region (Noor and Maniha 2020). **B** Binding of the host ACE-2 receptor with the SARS-CoV-2 spike protein leading toward the viral fusion within the host has been shown. Indeed, different conformations of the spike (S) RBD domain in opened and closed states function in this mechanism which has been elaborately discussed by Huang et al. (2020) (not shown in this diagram). **C** Amino acid alignment within the SARS-CoV-2 Spike (S) protein. The S protein contains (1) the extracellular N-terminus domain (amino acids 1–13), (2) the S1 subunit (14–685 residues), and (3) the S2 subunit (686–1273 residues): the fusion peptide (FP) (788–806 residues) plus the heptapeptide repeat sequence 1 (HR1) (912–984 residues) and HR2 (1163–1213 residues); a transmembrane (TM) domain (1213–1237 residues) across the viral membrane, one region for receptor binding and one for membrane fusion; and finally, an intracellular C-terminal domain (CTD/ cytoplasm domain (1237–1273 residues) (Noor and Maniha 2020). As stated earlier, the RBD situated in the S1 subunit binds to the cell host ACE2 receptor (Kahn and McIntosh 2005). Besides, FP is actually a short segment of 15–20 conserved hydrophobic amino acid residues (mostly glycine alanine), which mediates the anchoring of the target membrane when the S protein adopts the conformation (Huang et al. 2020). Moreover, this is noteworthy that the targeting the heptad repeat (HR) has attracted the greatest interest in therapeutic drug discovery so far (Noor and Maniha 2020)

In order to understand the effective actions of the vaccines for eliciting the required host immunity not only against the original Wuhan strain of SARS-CoV-2 but also against the emerging variants of concern (VOC) and the variants of interest (VOI), it is worth to understand how the viral RNA is replicated, the structural proteins are synthesized following their assembly and packaging in the host cell, and how the mature viruses are then released and propagated. Such life cycle of SARS-CoV-2 has been diagrammatically explained in the model shown in Fig. 1. This enveloped, positive-sense, single-stranded (ss) RNA virus consists of several structural proteins needed for invasion and the subsequent pathogenesis into the host cells. Once inside the cell the infecting SARS-CoV-2 RNA encodes (1) the envelope protein (E), spike or surface glycoprotein (S), membrane protein (M) and the nucleocapsid protein (N) which are required to make up the virus particles; and (2) the nonstructural proteins (nsps) which facilitate the virus assembly, transcription, replication and the utilization of the accessory proteins (Noor and Maniha 2020; Asaduzzaman et al. 2020). The spike (S) glycoprotein, residing outside of the virus particle, imparts the crown-like appearance of the virus (Noor and Maniha 2020). The most important thing to understand the viral virulence is to ponder that the spike (S) protein mediates the attachment of the virus particle and subsequent enter into the host cell; and it is to be noted that the S protein is the prime target for the vaccine action development and for the antibody-based therapies (Noor and Maniha 2020; Heinz and Stiasny 2021; Noor 2021a, c; Zhang et al. 2020; Huang et al. 2020). The S proteins are coated with polysaccharide molecules which apparently camouflage them and hence thus help the virus to escape the surveillance by the host immune system during the viral entry (Noor and Maniha 2020; Huang et al. 2020). The binding of the host angiotensin-converting enzyme 2 (ACE-2) by the S protein facilitates the viral entry into the host through the membrane fusion, resulting in the release of the viral RNA into the host cytoplasm (Figs. 1, 2). Once the S protein binds to the ACE-2 receptor, the trans-membrane serine protease 2 (TMPRSS2) in the host cell membrane facilitates the viral entry into the host cytosol by activating the S protein (Noor and Maniha 2020; Huang et al. 2020).

Compared to SARS-CoV-1 and MERS-CoV, SARS-CoV-2 is being noticed with much more high degree of transmissibility, absolutely demanding for an appropriate vaccine; and with a great hope, a number of vaccines are currently in use under the emergency authorized use (EAU) (Noor 2021a, c; Gómez et al. 2021; Forni and Mantovani 2021; Pushparajah et al. 2021; Yadav et al. 2021; Ura et al. 2021). However, the emerging variants of SARS-CoV-2 raised the questions about the efficacy of the currently used vaccines (Noor 2021c; Ura et al. 2021; Mascellino et al. 2021; Chakraborty et al. 2021a; Ferré et al. 2021; Wang et al. 2020; Muik et al. 2021; Shinde et al. 2021). After the wild type originated in Wuhan, China, the Alpha variant (of B.1.1.7 lineage) originated in the UK, Beta variant (B.1.351 lineage) originated in South Africa, Gamma variant (of P.1 lineage) originated in Brazil, Delta variant (B.1.617.2 lineage) in India; and Omicron variant (B.1.1.529 lineage) originated in South Africa (Chakraborty et al. 2021a; Ferré et al. 2021). Indeed, the mutated viruses are more likely to fit and to survive, resulting in more aggressive and transmissible phenotypes among the human community (Mascellino et al. 2021; Ferré et al. 2021). Along these lines, the present review emphasized the details of all the major vaccines currently administered into the global population as well as an inference about their effectiveness against the emerging variants.

## Main text

### Significance of spike (S) protein in the development of COVID-19 vaccines

**Fig. 3:**
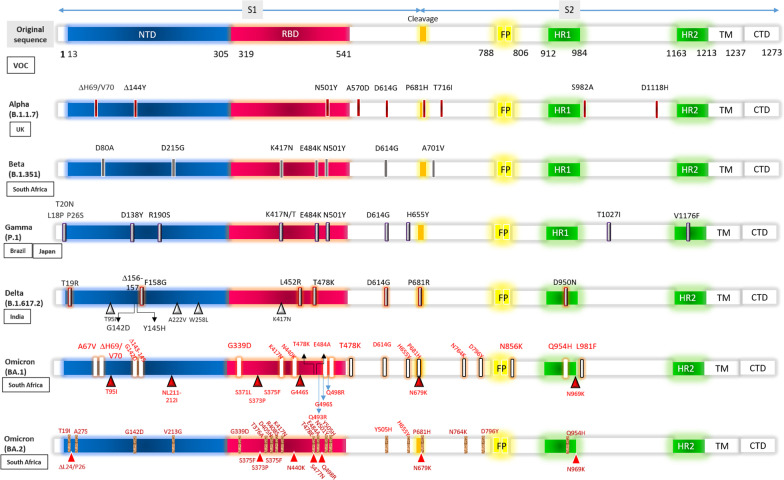
SARS-CoV-2 variants of concern (VOC). The amino acid changes have been shown in all the VOC which have been compared with the genome sequence of the spike (S) protein of the original SARS-CoV-2 strain from Wuhan, China. *NTD* N-terminal domain, *RBD* receptor binding domain, *FP* fusion peptide domain, *HR1* Heptad repeat 1, *HR2* Heptad repeat 2, *TM* transmembrane domain, *CTD* C terminal domain

Since the spike protein serves as the major target for most of the vaccines, and mostly the key mutations within the S protein generate the variants, it is important to understand its structural features and its function in order to extrapolate the mode action of vaccines (Noor 2021a, c; Zhang et al. 2020; Huang et al. 2020; Gómez et al. 2021; Forni and Mantovani 2021; Pushparajah et al. 2021; Keech et al. 2020; Tian et al. 2021). Figure 2 shows the structure of the SARS-CoV-2 S protein together with the binding mechanism of the hACE-2 receptor by the S protein, which resides as trimers, the crown-like structures surrounding the virus. The S1 and S2 subunits form the bulbous head and stalk region (Huang et al. 2020). The interaction between the receptor binding domain (RBD, which is the key site of mutations posed by the variants) within the S protein and hACE-2 facilitates the viral fusion (Huang et al. 2020; Noor 2020). Indeed, not only the RBD site but also the positions and the identity of the amino acids within the whole S protein are also important since the changes/shifting of amino acids along the entire length of S protein may cause the emergence of the viral variants as shown in Fig. 3 (Gómez et al. 2021; Mascellino et al. 2021; Chakraborty et al. 2021a; Ferré et al. 2021; Wibmer et al. 2021). COVID-19 vaccines that are in current use worldwide mainly target the SARS-CoV-2 spike protein with the aim of producing the neutralizing antibodies as well as eliciting the cell-mediated immunity (Noor 2021c; Gómez et al. 2021). Therefore, it is worth to analyze the vaccine efficacy against these variants.

### COVID-19 vaccines currently in use

Several vaccines have been manufactured so far with the nucleic acid (RNA and DNA) platform, adenovirus vector platform, the traditional platforms of vaccine preparation using inactivated or live attenuated viruses, protein subunit platform, adjuvant recombinant platform, nanoparticle-based vaccine platform and virus-like particle (VLP)-based platforms (Heinz and Stiasny 2021; Noor 2021a, c; Gómez et al. 2021; Forni and Mantovani 2021; Pushparajah et al. 2021; Yadav et al. 2021; Ura et al. 2021; Kyriakidis et al. 2021; Pollet et al. 2021). Based on the recent information, a list of the COVID-19 vaccines currently in use is given in Table 1. Based on the nucleic acid platform, the mRNA vaccines BNT162b2 manufactured by BioNTech (Mainz, Germany)/Pfizer (New York, USA) and mRNA-1273 developed by Moderna, Cambridge, MA, USA, have been found with 94% effectiveness (Heinz and Stiasny 2021; Noor 2021a, c; Gómez et al. 2021; Yadav et al. 2021). The adenovector vaccine ChAdOx1-S/AZD1222, manufactured by the University of Oxford/AstraZeneca, Cambridge, UK, showed around 70% efficacy after the first dose and 81.3% after the second (Heinz and Stiasny 2021). Another adenovirus vector vaccine, Sputnik V, developed by Gamaleya Institute in Moscow, was found with nearly 92% efficacy; and the Ad26.COV2.S vaccine from the Janssen Pharmaceuticals of Johnson & Johnson, Belgium, was found with nearly 70% efficacy (Heinz and Stiasny 2021).

**Table 1:** COVID-19 vaccines currently in use

Vaccine platform	Vaccine name	Composition	Mode of action	Efficiency	References
mRNA vaccines (mRNA encoding the vaccine antigen is encapsulated with a lipid-based carrier, injected into the host; and then using the cellular protein translation machinery the mRNA produces the appropriate antigen which in turn provokes host immune response)	BNT162b2 BioNTech (Mainz, Germany)/Pfizer (New York, USA)	Nucleoside-modified mRNA (uridines are completely replaced by N1-methylpseudouridines), encoding the sequence of the full-length S protein with two stabilizing proline mutations in S2. It contains lipid nanoparticles (LNP) for delivery. Storage at − 70 °C. Contains 30 µg RNA	mRNA encoding the S protein, encapsulated into an LNP, enters the host cell by endocytosis. After endosomal escape into the host cytosol, the S specific mRNA is translated along ribosomes associated with the endoplasmic reticulum (ER). The newly synthesized S protein is transported into the lumen of ER (as happens in case of natural infection). Further transport occurs via exocytic pathway leading to the expression of the spike protein at the plasma membrane. The S protein is also degraded and enters the major histocompatibility complex (MHC) I and II pathways	95% (Phase III clinical trials)	Heinz and Stiasny (2021), Noor (2021a, c), Gómez et al. (2021) and Yadav et al. (2021)
	mRNA-1273 (Moderna, Cambridge, MA, USA)	Nucleoside-modified mRNA (as stated above), encoding the sequence of the full-length S protein with two stabilizing proline mutations in S2. It contains LNP for delivery. Storage at − 20 °C. Contains 100 µg RNA		94.1% (Phase III clinical trials)	Heinz and Stiasny (2021), Noor (2021a, c), Gómez et al. (2021) and Yadav et al. (2021)
Adenovector vaccines (using adenoviruses as vectors based on their capacity to induce potent innate and adaptive immune responses)	Sputnik V (Gamaleya Institute in Moscow)	Number of viral particles, i.e., the human adenovirus 5 (hAd5) plus hAd26: 1 × 10^11^ (18 µg of adenovirus protein; based on approximately 100 million amino acids composing adenovirus capsid)	Adenovector containing S gene as part of the viral DNA enters host cytosol (endocytosis). After escaping endosomal lysis, capsid migrates into the nucleus and produces S-specific mRNA transcripts. The S specific mRNA is translated in ribosomes associated with the ER, and the S protein is transported into the lumen; exocytosis occurs leading to the expression of S protein at the plasma membrane. The S protein is also degraded and enters MHC I and II pathways	91.6% (Phase III clinical trials)	Heinz and Stiasny (2021)
	ChAdOx1-S/AZD1222 (University of Oxford/AstraZeneca, Cambridge, UK)	Uses a chimpanzee common cold viral vector (ChAdOx1), which delivers the code that allows the host cells to make the SARS-CoV-2 spike protein Number of viral particles: 5 × 10^10^ (9 µg of adenovirus protein; based on approximately 100 million amino acids composing adenovirus capsid)		70.4% after the 1st dose and 81.3% after the 2nd dose (Phase III clinical trials)	Heinz and Stiasny (2021)
	Ad26.COV2.S (Janssen Pharmaceuticals [pharmaceutical company of Johnson & Johnson], Beerse, Belgium)	The Janssen hAd26 vaccine contains stabilizing mutations similar to those engineered into the mRNA vaccines	Humoral immune responses (binding and neutralizing antibody responses), cellular immune responses (CD4^+^ and CD8^+^ T cell responses), a variety of antibody subclasses, Fc receptor binding properties, and antiviral functions were noticed	66.9% (Phase III clinical trials); another Phase III clinical trial is ongoing (NCT04436276)	Heinz and Stiasny (2021), Gómez et al. (2021) and Stephenson et al. (2021)
Inactivated whole-virus vaccine	CoronaVac/ Pi PiCoVacc (Sinovac Biotech, Beijing, China) and BBIBP-CorV (Sinopharm, Beijing, China)	The virus is grown in Vero cells, chemically inactivated by β-propiolactone (BPL) followed by purification; and then supplemented with adjuvants. The inactivated coronaviruses can no longer replicate although their proteins, including spike, remain intact	PiCoVacc elicited the SARS-CoV-2-specific neutralizing antibodies in mice, rats and non-human primates. The antibodies neutralized 10 representative strains. Immunizations using 3 μg or 6 μg per doses imparted partial or complete protection in macaques against viral challenge, respectively	86% (China, Bahrain, UAE); 78% (Brazil), 91.25% (Turkey), 65.3% (Indonesia)	Heinz and Stiasny (2021), Ura et al. (2021) and Gao et al. (2020)
	Covaxin (Bharat Biotech, Hyderabad, Telangana, India)	The vaccine is based on an influenza virus where gene sequences from SARS-CoV-2 are inserted into M2SR-vaccine platform (M2-ion channel protein-deficient single replication)	Covaxin works by directing the immune system to elicit antibodies against the SARS-CoV-2, especially, to the so-called spike (S) proteins	India authorized Covaxin on January 2021 although no Phase III results were shown	Heinz and Stiasny (2021), Kyriakidis et al. (2021) and Corum and Zimmer (2021)
Subunit vaccine	NVX-CoV2373 (Novavax, Gaithersburg, MD, USA)	The antigenic part is a recombinant full-length S protein with stabilizing mutations produced in Sf9 insect cells. The S protein is extracted by detergent solubilization and chromatographically purified. Nanoparticles are formed by mixing the purified protein with saponin (adjuvant), cholesterol and phospholipid. Storage at 4 °C	NVX-CoV2373 S form 27.2-nm nanoparticles are thermostable and bind with high affinity to hACE2 receptor. In mice model, NVX-CoV2373 with saponin-based Matrix-M adjuvant elicit high titer anti-S IgG that blocks hACE2 receptor binding, and neutralize virus; and also induces CD4^+^ and CD8^+^ T cells, CD4^+^ follicular helper T cells (Tfh), and antigen-specific germinal center (GC) B cells in the spleen. In baboons, high titer anti-S antibodies and antigen-specific T cells were also noticed	89.1% (Phase III clinical trial in UK) and 60.1% in South Africa^a^	Heinz and Stiasny (2021), Tian et al. (2021) and Wadman and Cohen (2021)
				Another Phase 1/2 clinical trial is also ongoing (NCT04368988)	Tian et al. (2021)

^a^Such difference may be associated with antigenic differences between the circulating Alpha and Beta variants

The inactivated whole-virus vaccine CoronaVac/PiCoVacc, manufactured by Sinovac Biotech, Beijing, China; and BBIBP-CorV, developed by Sinopharm, Beijing, China, were noticed with a fluctuating efficacy of 65–86% in different target populations from different geographic locations (Heinz and Stiasny 2021; Ura et al. 2021; Gao et al. 2020). Another inactivated whole virus vaccine, Covaxin, developed by Bharat Biotech, India, has been authorized for the mass administration in India on January 2021 (Heinz and Stiasny 2021; Kyriakidis et al. 2021; Corum and Zimmer 2021). The Phase III clinical trials with nanoparticle-based subunit vaccine NVX-CoV2373, manufactured by Novavax, Gaithersburg, USA, have shown 89.1% effectiveness in the UK, whereas the effectiveness reduced 60.1% in South Africa, revealing the emergence of variants, i.e., the variant 501Y.V2 consisting of the B.1.351 lineage (i.e., the Beta variant) which might neutralize the action of the vaccine (Heinz and Stiasny 2021; Tian et al. 2021).

### Mutations with the S protein and emergence of SARS-CoV-2 variants

The emerging variants of SARS-CoV-2 have been notified as either the VOCs including (1) Alpha (20I/501Y.V1 variant of B.1.1.7/ lineage), (2) Beta (20H/ 501Y.V2 variant of B.1.351 lineage), (3) Gamma (20J/501Y.V3 variant of P.1 lineage), (4) Delta variant of B.1.617.2 lineage, and (5) the Omicron variant of B.1.1.529 lineage; or the variants of interest (VOIs) including (1) Eta (B.1.525 lineage, New York, USA), (2) Iota (B.1.526 lineage, New York, USA), (3) Zeta (P.2 lineage, Brazil), and (4) Epsilon (B.1.427/B.1.429 lineages) (Chakraborty et al. 2021a; Ferré et al. 2021). The evolutionary patterns, geographical distributions, transmission patterns and the mutational event diversity of all these emerging variants have been extensively analyzed; and the transmission pattern was found to be the highest in case of the Alpha variant of B.1.1.7 lineage (Mascellino et al. 2021). Lots of reports stated that mutations in the S protein are of significant concern since the vaccines have been principally designed to elicit antibodies against components of the S protein (Mascellino et al. 2021; Chakraborty et al. 2021a).

Indeed, five VOCs based on their clinical severity and transmission potential due to critical mutations/amino acid changes (both shifting/replacement and deletion) on the global public health have been identified till date (Noor 2021a; Gómez et al. 2021; Yadav et al. 2021; Mascellino et al. 2021; Ferré et al. 2021). The emerging SARS-CoV-2 variants have been examined, and high frequency of mutations within the S protein (Fig. 3) have been noticed which in turn may provoke the structural changes hindering the vaccine effectiveness (Zhang et al. 2020; Gómez et al. 2021; Yadav et al. 2021; Ura et al. 2021; Mascellino et al. 2021; Chakraborty et al. 2021a; Ferré et al. 2021). Among the variants examined from the different SARS-CoV-2 strains of different geographical locations, the UK variant (Alpha), the South African variant (Beta), the Brazilian variant (Gamma), the Indian variant under investigation (VUI-202012/01) of B.1.1.7 lineage (i.e., Alpha variant), the Indian VOC Delta variant of B.1.617.2 lineage, the current South African VOC Omicron variant of B.1.1.529 lineage, the VOCs Mu of B.1.621 lineage (emerged in Colombia in early 2021) and Lambda of C. 37 lineage (emerged in Peru in late 2020), and the former VOI in California namely Epsilon variant of B.1.427/B.1.429 lineage are of clinical significance (Noor 2021a; Gómez et al. 2021; Yadav et al. 2021; Ferré et al. 2021). As stated elsewhere, the mutations may alter the interaction of the S protein or the RBD within the S protein which is supposed to be connected to the hACE2 receptor, thereby facilitating the escape of the action of the vaccines implemented as well as refashioning the viral infection rate (Zhang et al. 2020; Gómez et al. 2021). Besides, such an altered interaction of SARS-CoV-2 with the ACE2 receptor may also facilitate the host shifts, thereby increasing the viral transmissibility (Gómez et al. 2021). After the beginning of the COVID-19 pandemic, a variant was found with a single D614G mutation (mutation in the aspartic acid residue) in the spike (S) protein, increasing the transmissibility in Europe in the early February (Zhang et al. 2020; Ura et al. 2021; Mascellino et al. 2021). The transmissibility of the G614 genotype (mutation in the glycine residue in 614th position) increased up to 70% by May 2020 (Zhang et al. 2020). The continuous mutation within the SARS-CoV-2 S protein (Fig. 3) is an emerging concern regarding the vaccine efficiency which is currently in use (Heinz and Stiasny 2021; Noor 2021a, c; Forni and Mantovani 2021; Yadav et al. 2021; Ura et al. 2021).

### The Alpha and Beta variants

The mutations within the most important antigenic sites (i.e., the S protein) have been appearing to render the virus more transmissible, of which the UK Alpha variant has 14 mutations in the RBD site and three amino acid deletions increasing the viral transmissibility by 50%; the South African Beta variant and the Brazilian Gamma variants, both having two additional mutations causing significant changes within the epitopes of the protein surfaces which are relevant for antibody recognition (E484K and K417N/K471T, respectively), are remarkable (Heinz and Stiasny 2021; Gómez et al. 2021; Yadav et al. 2021). This is noteworthy that three mutations; i.e., H69-V70del, N501Y and P681H of B.1.1.7 (Alpha variant), are significant in terms of viral infectivity and transmission potential (Gómez et al. 2021). Moreover, both the Alpha and Beta variants share the N501Y mutation within the RBD domain of S protein (Fig. 3) which confers an increased binding affinity of the spike RBD for the hACE2 receptor, resulting in elevated level of viral transmission and host protective immunity avoidance (Gómez et al. 2021).

### The Delta variant

Migration of Alpha variant which was initially termed as VUI-202012/01 was noticed in India by the Indian travelers returning from South Africa (Johannesburg) and Tanzania (Yadav et al. 2021). The Indian Delta variant comprises double mutation whereby (1) glutamate has been replaced by glutamine at the 484th spot of spike protein (denoted by E484Q) and (2) the substitution of leucine with arginine has taken place at the 452 position (denoted by L452R). Such mutations, especially the E484Q, may facilitate the virus more capable of binding the host ACE-2 receptors by the spike protein. L452R imparts viral replication and can evade antibodies (Ferré et al. 2021). It is to be noted that such RBD mutations L452R and E484Q along with P681R in the furin cleavage site possibly resulted in an augmented ACE2 binding as well as increasing the rate of S1-S2 cleavage, thereby facilitating enhanced transmissibility (Gómez et al. 2021).

### Emergence of Omicron variant

The first case of Omicron variant (B.1.1.529 lineage) of SARSCoV-2 was confirmed on November 9, 2021, reported by WHO on November 24, 2021, from South Africa and Botswana and classified as VOC which carries mutations found in other VOCs (Saxena et al. 2021; Wang et al. 2021; Collie et al. 2021; Barda et al. 2021; Poudel et al. 2021). More than 50 mutations have been identified in the recent Omicron variant of which 50% is within the RBD site of the S protein (Fig. 3), i.e., 26–32 substitution, deletion and insertion mutations only on the S protein (Ferré et al. 2021; Poudel et al. 2021). Only a dozens of these mutations have been identified in the past (Poudel et al. 2021). The Omicron variant has the deletion at spike position 69–70, which is similar to the Alpha variant; there are three key mutations conferring immune escape which have been also noticed in the Beta and Gamma variants (Poudel et al. 2021). Moreover, at least 15 critical mutations in the S protein as identified by the Center for Disease Control (CDC) possibly imparted the viral infectivity advantages especially over the fatal Delta variant (B.1.617.2) (Wang et al. 2021; Poudel et al. 2021). A closer notification of a cluster of mutations at the S1–S2 furin cleavage site and a combination of mutations in the RBD of the S protein has made this variant more fatal than others; and these mutations have been also identified in the Delta variant (Ferré et al. 2021; Poudel et al. 2021). It is noteworthy that the Alpha variant has accumulated 23 mutations and the Gamma variant contains 17 non-synonymous mutations (Fig. 3) which reveal increased numbers in the Omicron variant rendering it more fatal (Gómez et al. 2021).

### Health risks posed by the variants

Indeed, the variants of concern (VOC) are likely to pose potential risk to human health. Despite the viral proofreading exonuclease activity, multiple variants emerged with a benefit on virus replication and dissemination potential resulting in large incidence of infection rates, augmenting the host immune protection system as has been noticed from the dominant Alpha variant to the current Omicron variant (Gómez et al. 2021). The Delta variant of B.1.617.1 lineage may instigate increased body weight loss, intense viral load in lungs and significant lung lesions. Besides the high transmission potential, the clinical consequences are principally the higher rate of damage of major organs, morbidity and mortality by the variants compared to that of Wuhan’s reference SARS-CoV-2 strain, i.e., wild type (Gómez et al. 2021; Saxena et al. 2021).

### Assessment of vaccine efficacy against the major SARS-CoV-2 variants

The mRNA-1273 vaccine, mRNA-BNT162b2 vaccine, ChAdOx1-S/ AZD1222 vaccine, JNJ-78436735 vaccine and NVX-CoV2373 vaccines are so far widely used commercial vaccines (Heinz and Stiasny 2021; Pushparajah et al. 2021; Kyriakidis et al. 2021). Against the Alpha variant, both BNT162b2 (Pfizer) and mRNA-1273 (Moderna) were found to decrease the neutralizing antibodies (similar effects were noticed in case of P.1 and B.1.351 variants as well), while NVX-CoV2373 (Novavax) was found to impart 85.6% efficacy in the UK population, whereas 60% efficacy was noticed in the South African population (Chakraborty et al. 2021a; Sanches et al. 2021; Chakraborty et al. 2021b). Against B.1.351 variant (N501Y.V2 lineage, consisting of three receptor-binding domain mutations and five additional N-terminal domain mutations), Ad26.COV2.S vaccine (Johnson & Johnson) showed 64.0% efficacy in Brazilian population and 52% efficacy in the South African population; and NVX-CoV2373 (Novavax) showed 49% efficacy in the South African population (Chakraborty et al. 2021a). The BNT162b2 vaccine (Pfizer/BioNTech) has been found to be effective against the P.1 variant although this variant can evade inhibition by neutralizing antibodies (Ura et al. 2021). The effectiveness of Pfizer-BioNTech, Moderna, Oxford/AstraZeneca and Sinovac vaccines against both the gamma and delta variants has been scored as 85%, 78%, less than 70%, and 66%, consecutively (Cevik et al. 2021). Two doses of Pfizer’s BioNTech COVID-19 vaccine (BNT162b2) have been found to cross-neutralize some of the circulating Delta variants, while the effectiveness of Johnson and Johnson vaccine was noticed to be reduced from 66.9 to 60% against this variant (Mascellino et al. 2021). Compared to BioNTech- and the Johnson and Johnson vaccines, the Moderna vaccine has been noticed with an efficacy of approximately 94.1% against the Delta variant, while the Pfizer and AstraZeneca vaccines were less effective against this variant than that of the Alpha variant (Mascellino et al. 2021; Thye et al. 2021).

While the Alpha variants were neutralized by the Biontech/Pfizer mRNA vaccine and the Moderna mRNA vaccines, the effectiveness of the Oxford/Astra Zeneca vaccine was noticed to be ninefold lower than those of the RNA vaccines (Heinz and Stiasny 2021; Muik et al. 2021). The Beta variants showed the vaccine immunization escaping strategy in several cases using convalescent plasmas (Wibmer et al. 2021). The lower efficacy rates of (1) the Novavax subunit vaccine (Phase III trial) in case of Beta variants than those of the Alpha variant; and of (2) the Janssen Adeno26 (Ad26.COV2.S) vaccine in Latin America and South Africa than in the USA also project the influence of mutation (resulting in the circulating variants) on the vaccine efficiency (Heinz and Stiasny 2021). A phase III randomized and placebo-controlled trial of the single-shot Ad26.COV2.S is ongoing using 40,000 participants, and primarily the efficiency of the vaccine has been estimated as 66.9% (Gómez et al. 2021).

### Efficacy of the current vaccines against the variants, and the need for booster

According to the experimental demonstration, it is not unlikely that VOCs may withstand the neutralizing action of immune sera induced by the currently used vaccines including the Pfizer, Moderna, AstraZeneca, Janssen, Sputnik, Novavax, Sinovac and others (Gómez et al. 2021). For example, in the Alpha variant of B.1.1.7 lineage 2.7–3.8-fold reduction was noticed in case of Moderna or Pfizer vaccines (1.8-fold–twofold) (Gómez et al. 2021; Muik et al. 2021; Wang et al. 2021). Vaccine efficacy against the Beta variant of B.1.351 lineage was worse than that of the Alpha variant (Gómez et al. 2021; Wang et al. 2021). The Pfizer vaccine decreased its efficacy from 94 to 64% against the Delta variant of B.1.617.2 lineage (Mascellino et al. 2021). The vaccine effectiveness against the omicron variant (of B.1.1.529 lineage) was noticed to be of 70% in South Africa (during the proxy omicron period), whereas the effectiveness was found to be significantly different from that during the comparator period, i.e., approximately 93% against hospitalized COVID-19 patients infected by the Delta variant of B.1.617.2 lineage (Collie et al. 2021). This revealed the reduction in vaccine effectiveness against the omicron variants. Such a decrease in vaccine effectiveness can be complimented by the administration of a booster dose of vaccine (Barda et al. 2021). Moreover, the study by Wang et al. (2021) showed that the huge number of mutations within the Omicron variant resulted in significant reduction in the viral S protein neutralization against human convalescent sera infected by natural COVID-19 infection, which actually exceeded all the other current VOCs and VOIs (Barda et al. 2021). Notably, an increased tendency of the host immune escape was noticed by this variant which is alarming for the current omicron infection (Wang et al. 2021). However, the efficacy of the current vaccines is clearly visible worldwide taking into consideration that the clinical trials are still in process, and the reason for developing the vaccines is to reduce the severity of the disease symptoms develop and to reduce the number of hospitalizations and urgent care visits. Therefore, booster vaccines are required which may lengthen the duration of the neutralized antibodies.

A recent study assessed the humoral memory response in 87 individuals within 1.3 and 6.2 months after SARS-CoV-2 infection with SARS-CoV-2, whereby the IgM and IgG titers against RBD of the S protein were found to decrease over time (Gaebler et al. 2021). In contrast, the RBD-specific memory B cells were found to be unchanged in quantity after 6.2 months after infection together with a surprising trait of clonal turnover, somatic hypermutation, resistance to RBD mutations and an increased potency (Gaebler et al. 2021). Thus, the memory B cell response against the virus keeps consistent with the viral antigen persistence. Moreover, the vaccine effectiveness has also been noticed not only in regards to such memory B cells (eliciting the neutralizing antibodies) but also from the long-lasting protection from hospitalizations, possibly due to the adaptability of B cells and T cells through vaccination against the emerging variants (Rubin 2021; Noor 2022). Such an effectiveness may be maintained by optimizing vaccine schedules and boosters along with the implications of the non-pharmaceutical interventions, like the wearing masks, social distancing, etc. (Rubin 2021; Diseases 2022). Moreover, while the double-dose vaccine-induced immune protection may be escaped by the Omicron variant, it can be mitigated by the booster vaccine dose (Ai et al. 2022). The recent work by Ai et al. (2022) on the post-booster vaccination revealed positive neutralization of the Omicron variants in 100% samples, thereby indicating that even the Omicron variant can escape the immunity elicited by the vaccine doses, vaccine-induced immune protection may be escaped by the Omicron variant which can be further neutralized completely by the booster dose administration (Ai et al. 2022).

## Conclusions

The present review briefly emphasized the major mutations within the SARS-CoV-2 spike (S) proteins and discussed the mode of actions of the currently used vaccines together with their efficacy against the mutant strains. During the ongoing third wave of COVID-19 caused by delta and mostly by the omicron variants, wide-ranging studies of the SARS-CoV-2 genomes are essential for the constant surveillance of the mutations within the viral spike protein or anywhere else, which would augment the development of appropriate vaccine. Genomic study and continuous surveillance of the emerging VOC and VOI of SARS-CoV-2 would augment the predictive epidemiology, the detailed genomic evolution raising the infectivity and transmissibility of the variants; and the host protective immune response can be examined as well. The sustainability of memory B cell responses against the viral re-infection is highly suggestive of eliciting a rapid and effective response to the viral re-infection, which in turn infers the effectivity of the current COVID-19 vaccines. However, as the protective measure, the efficacy of the vaccines would be pointed accurately based on the clinical trials against the emerging variants like the highly mutated Omicron variant; and such a study would be very much fruitful to ponder the advancement in vaccine development to establish the robust immunity against the SARS-CoV-2 variants. Moreover, looking at the data of the fully vaccinated and boosted population would be helpful to infer the effectiveness of the vaccine’s booster.

## Data Availability

Not applicable.

## Abbreviations

ACE-2: Angiotensin-converting enzyme-2
APCs: Antigen processing cells
ARDS: Acute respiratory distress syndrome
COVID-19: Coronavirus disease 2019
CTD: C terminal domain
DCs: Dendritic cells
E: Envelope
FP: Fusion peptide domain
GM-CSF: Granulocyte–macrophage colony-stimulating factor
HE: Hemagglutinin esterase
HR1: Heptad repeat 1
HR2: Heptad repeat 2
IFNs: Interferons
IL: Interleukin
IRF: Interferon regulatory factor
M: Membrane
MDA-5: Melanoma differentiation associated gene-5
MERS-CoV: Middle East respiratory syndrome coronavirus
MHC: Major histocompatibility complex
N: Nucleocapsid
NF-kB: Nuclear factor kappa light chain enhancer of activated B cells
NTD: N-terminal domain
RBD: Receptor binding domain
RIG-I: Retinoic acid-inducible gene I
S: Spike
SARS-CoV-2: Severe acute respiratory coronavirus 2
Tc: T cytotoxic cells
Th: T helper cells
TLRs: Toll-like receptors
TM: Transmembrane domain
VLP: Virus-like particle
